# Artificial intelligence in emergency department triage: perspective of human professionals

**DOI:** 10.3389/fdgth.2025.1693060

**Published:** 2026-01-06

**Authors:** Alina Petrica, Adina Maria Marza, Claudiu Barsac, Andreea Cebzan, Ioan Dragan, Daniela Zaharie, Raluca Horhat, Diana Lungeanu

**Affiliations:** 1 Department of Surgery, “Victor Babes” University of Medicine and Pharmacy, Timisoara, Romania; 2 Emergency Department, “Pius Brinzeu” Emergency Clinical County Hospital, Timisoara, Romania; 3 Emergency Department, Emergency Clinical Municipal Hospital, Timisoara, Romania; 4 Clinic of Anaesthesia and Intensive Care, “Pius Brinzeu” Emergency Clinical County Hospital, Timisoara, Romania; 5 Faculty of Mathematics and Computer Science, West University, Timisoara, Romania; 6 Department of Computer Science, West University, Timisoara, Romania; 7 Center for Modeling Biological Systems and Data Analysis, “Victor Babes” University of Medicine and Pharmacy, Timisoara, Romania

**Keywords:** AI attitude, AI literacy, AI-driven triage, education, emergency room triage, healthcare providers, human factors, IT professionals

## Abstract

**Background:**

The triage process in emergency departments (EDs) is complex, and AI-based solutions have begun to target it. At this pivotal stage, the challenge lies less in designing smarter algorithms than in fostering trust and alignment among medical and technical stakeholders. We explored professional attitudes towards AI in ED triage, focusing on alignments and misalignments across backgrounds.

**Methods:**

An anonymous online cross-sectional survey was distributed through professional networks of healthcare providers and IT professionals, between May 2024 and February 2025. The questionnaire covered four areas: (a) the General Attitudes towards Artificial Intelligence Scale (GAAIS); (b) professional background and career level; (c) challenges and priorities for AI applications in triage; and (d) the AI Attitude Scale (AIAS-4). Constructs from the extended Unified Theory of Acceptance and Use of Technology (UTAUT2) were also applied. Cluster analysis (*KMeans*) was conducted based on GAAIS-positive, GAAIS-negative, and AIAS-4 scores.

**Results:**

From a total of 151 professionals, *Kmeans* identified three clusters: K0 (cautious/critical, *n* = 39), K1 (enthusiastic/optimistic, *n* = 35), and K2 (balanced/pragmatic, *n* = 77). Approximately two-thirds of K2 (47/77; 61%) were healthcare providers. Six out of 20 (30%) medical professionals in K0 reported that AI could play no role in ED triage, but only 1/15 (7%) and 1/47 (2%) of healthcare providers gave this response in K1 and K2, respectively. Lack of knowledge of AI tools was also most frequent in K0 (14/39; 36%). Recognition of necessity of constraints showed marked contrasts in their mea*n* ± SD scores: (a) for data availability/quality, 2.95 ± 1.98 (K0), 4.27 ± 1.1 (K1), and 4.20 ± 0.94 (K2); (b) for the integration of AI-based applications into existing workflows, 2.95 ± 1.05, 4.20 ± 0.94, and 3.47 ± 1.02 in K0, K1, and K2, respectively. Among the UTAUT2 constructs, hedonic motivation differed most significantly, with mean ± SD values of 3.41 ± 1.0 (K0), 6.86 ± 0.97 (K1), and 5.07 ± 1.08 (K2).

**Conclusions:**

Stakeholders' perspectives on AI in ED triage are heterogeneous and not solely determined by professional background or role. Hedonic motivation emerged as a key driver of enthusiasm. Educational strategies should follow two directions: (a) structured AI programs for enthusiastic developers from diverse fields, and (b) AI literacy for all healthcare professionals to support competent use as consumers.

## 1 Introduction

Artificial intelligence (AI) is increasingly present in society and it has also been successfully leveraged to improve medical decision making (1). While many healthcare professionals welcome AI-based solutions aimed at improving clinical work, others are more cautious, even skeptical, about its widespread use in daily clinical practice (2–6).

Emergency department (ED) triage is a complex task and a crucial decision point in emergency medicine. Its overcrowding is a growing global challenge, fueled by increased admission delays and patient influx, leading to prolonged ED length of stay (ED-LOS), treatment delays, decreased patient satisfaction, and poor outcomes. Validated triage systems, such as the Emergency Severity Index (ESI), the Canadian Triage and Acuity Scale (CTAS) or the Manchester Triage Scale (MTS), are not only administrative: they have demonstrated strong predictive value for admission, resource utilization, and mortality (7–9).

Nevertheless, there is room for improvement and AI-based solutions have already targeted emergency triage (8, 10–20); the literature emphasizes existing or future benefits in disease detection and prediction (regarding chief complaint, level of emergency, or clinical outcome), actual triage practice (e.g., improving AI-assisted ED triage, e-triage, or home-based patient triage), and human autonomy (e.g., disease tracking, activity detection, and mobile health) (12, 14, 17, 18, 21–25). Triage time in particular, defined as the time between ED check-in and triage code assignment, is frequently prolonged during peak hours, when patient volume increases dramatically and triage nurses may inadvertently overlook the most urgent cases; AI-powered solutions have been shown to be beneficial (14, 17–20). Despite these reported successes, some authors have expressed concerns about issues such as AI applications trained for single tasks, thus performing poorly in complex situations, i.e., better than lay individuals, rather than healthcare providers; such issues involve ethical and liability concerns, so these authors recommend using the AI-driven applications only in conjunction with human professionals who are expected to assume responsibility (10, 11, 13, 22, 24). At the same time, healthcare prompt engineering is developing (22, 23, 25), and promising high-performance AI-driven systems are integrating medical records, multimodal imaging, and medical literature (3, 4, 26).

However, AI in ED triage is not just a new tool, and its applications are still underexplored beyond the performance of algorithms. The adoption of AI-supported triage could mark a turning point, forcing the ED professionals to reexamine their positions, moving from being sole clinical decision-makers to roles such as team leaders collaborating with digital tools. At this pivotal stage of medical AI, many acknowledge that the real challenge is not developing the smartest algorithm, but cultivating the right mindset, trust, and cultural environment for AI to enhance—rather than degrade—the art of emergency care (2, 10, 12, 27).

While AI-based solutions in healthcare and medical decision-making are developed by computer scientists or other technical experts, medical professionals play an important role as consumers, facilitators, and even developers (i.e., as leaders participating in the development of digital tools) (15, 28–32). The overlapping in visions and expectations of these stakeholders has received little attention, even though it could be crucial to the success of the AI-driven solutions and their subsequent implementation.

We conducted an anonymous cross-sectional survey of medical and technical professionals to gather their perspectives on AI in ED triage. Our investigation aimed to explore: (a) the opinions and attitudes of professionals with various roles (i.e., consumers, facilitators, developers) towards AI in ED triage; (b) the (mis-)alignments in perspectives among these professionals; and (c) potential common paths for development in AI tools and AI-competent human resources, namely identifying priorities in professionals' perspective.

## 2 Materials and methods

### 2.1 Study design, participants and data collection

An anonymous online cross-sectional survey (implemented by Google Forms) was distributed via professional networks and specifically targeted relevant respondents: healthcare providers (medical doctors and nurses), advanced medical students (after their fourth year course of emergency medicine), computer engineers, computer scientists, programmers, advanced students in computer science and computer engineering. Snowball sampling approach was employed: potential respondents in Romania were targeted and invited to further recruit other fellow professionals (regardless of their country, nationality, or place of residence), rather than acquaintances from the general population. This type of non-probability sampling was deemed acceptable given the survey's objectives, which focused on exploratory investigations rather than hypothesis testing; therefore, no prior sample size calculation was performed.

The questionnaire was administered in English, as a universal professional language, so there was no need to translate emergent AI terminology and no language barriers in sampling. Data collection began only after participants confirmed informed consent through a mandatory initial item.

The study was conducted in accordance with the Declaration of Helsinki. Ethics Committee approval was waived due to the anonymous and professionally targeted nature of the questionnaire.

A panel of experienced professionals in emergency medicine and computer science designed the survey questionnaire based on the research objectives, related reviews and expert opinion articles (2, 5, 8–12, 14, 20), and their own professional expertise. A Delphi technique was applied to reach expert consensus and to determine: (i) the main contributors to the perspective on AI in ED triage; (ii) the relevant topics, and metrics to assess or gauge in order to achieve the objectives (i.e., identify alignments or misalignments in perspectives).

The questionnaire comprised four areas: (a) the General Attitudes towards Artificial Intelligence Scale (GAAIS); (b) information on professional background, and education/ profession level (three levels, i.e., undergraduate, junior graduate, and senior professional); (c) questions on challenges and priorities for future AI applications in the triage process; and (d) the AI Attitude Scale (AIAS-4). Both AI-related attitude scales had been previously validated and published (33–36). The face validity of the triage-specific questions (i.e., their relevance to this project) was established through the aforementioned Delphi technique during their development; no prior formal validation was performed for these items. All questions were mandatory. To compensate for possible common method bias (37), AI-related scales were included at the beginning and end of the questionnaire, with the sections of factual questions and ED triage questions between them. In addition, related topics were addressed in non-adjacent questions, and different question formats were alternated (such as, checkboxes, linear scales, and multiple choice grids).

The General Attitudes towards Artificial Intelligence Scale (GAAIS) comprises 20 items measured on a 5-point Likert scale, considering two factors: positive (12 items) and negative (8 items) components (33, 34). It has a broad scope, assessing general beliefs about AI with psychological references, including positive/ negative perceptions, risk/ opportunity evaluations, and emotional responses.

The AI Attitude Scale (AIAS-4) consists of four items measured on a 10-point proportional scale, considering a single factor (35, 36). It focuses more specifically on future-oriented attitudes towards AI and complements the GAAIS.

The triage-specific questions employed the following measurement tools: (a) 5-point Likert scales; (b) ranking scales imposing a 5-point hierarchy in the items provided; (c) checkboxes; (d) open-ended questions for free-text comments. This report presents the analysis of the quantitative data collected using the questionnaire.

To enable comparisons of the perspectives and attitudes identified in this investigation with studies conducted using various research approaches, the constructs of the extended Unified Theory of Acceptance and Use of Technology (UTAUT2)—namely, performance expectancy, effort expectancy, social influence, facilitating conditions, hedonic motivation, and price value (38, 39)—were also applied to interpret the findings of this project. UTAUT2 is based on the paradigm of technology acceptance and incorporates previous theories; its reliability has been validated (40, 41). UTAUT2 is widely used in studies on acceptance of, or attitudes towards, technologies such as digital health, telemedicine, web-based technology, or blockchain technology (41, 42).

**Figure 1:**
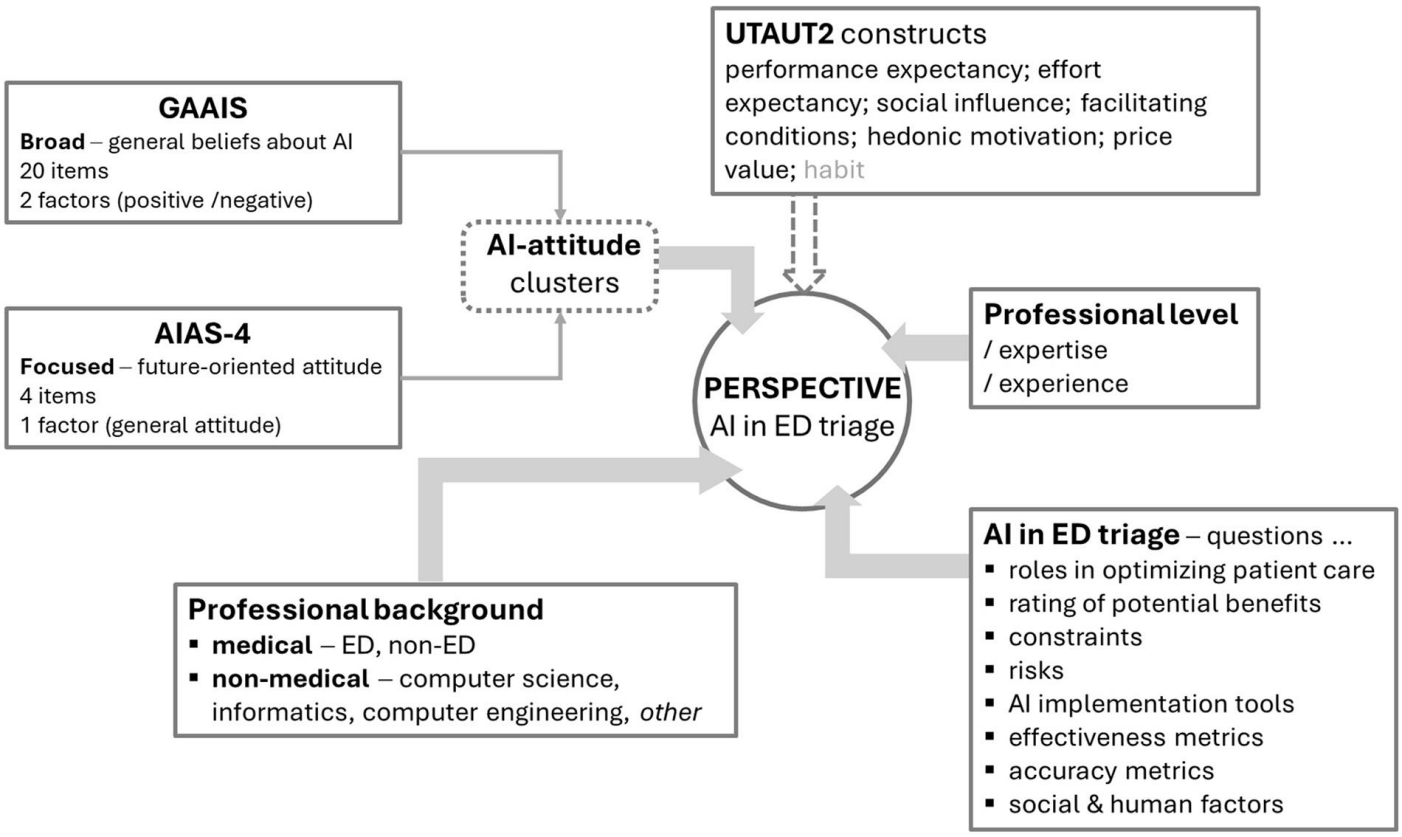
Study diagram with the factors shaping professionals’ perspective on AI in ED triage. AIAS-4, 4-item AI attitude scale; ED, emergency department; GAAIS, general attitudes towards artificial intelligence scale; UTAUT2, extended unified theory of acceptance and use of technology.

Figure 1 presents a schematic overview of the factors shaping professionals' perspectives, as targeted in our survey. The full questionnaire of this study is openly provided on *figshare* with the curated data file https://doi.org/10.6084/m9.figshare.30847343.

### 2.2 Data analysis

Reliability of the scale measurements was assessed using Cronbach's alpha, calculated for each scale and subscale. Values above 0.7 were interpreted as indicating moderate internal consistency. The intraclass correlation coefficient (ICC) with a 95% confidence interval (CI) was also computed to evaluate measurement reproducibility, with values greater than 0.5 considered evidence of moderate to good reproducibility.

Data analysis followed an exploratory approach without formal statistical hypotheses. Descriptive statistics included observed frequency counts and percentages for categorical variables, and means with standard deviations (SD) for numerical variables, regardless of distribution. Data visualization employed column diagrams, heatmaps, and radar plots with aggregated metrics.

To examine differences in professional attitudes towards AI use in ED triage, respondents were grouped into clusters based on the three attitude-related subscales: GAAIS-positive, GAAIS-negative (reverse-scored items were inverted to align directionality), and AIAS-4. This approach aimed to identify distinct perspectives and further explore respondents' perspective in relationship with their backgrounds, experience, and ED triage–related answers (i.e., opinions or priorities). All three scorings were rescaled to a 0–1 range using min–max normalization, then *KMeans* clustering was performed using these normalized scorings. The *silhouette* score (43) was used to evaluate clustering quality. Clusters were visualized using principal components analysis (PCA), and subsequently applied as a grouping variable in downstream analyses regarding ED triage, including: preferences for AI tools; perceived risks, constraints, and benefits; priorities in evaluation metrics; and alignment with UTAUT2 constructs.

Data analysis was performed with the statistical software IBM SPSS v. 26 and R v. 4.4.3 packages.

### 2.3 Declaration of generative AI and AI-assisted technologies

In the research process, *ChatGPT-5* from *Open AI* was used as research assistant to analyze the data. Authors checked the generated R code and all results, and they assume full responsibility and accountability for the contents of the work.

*ChatGPT-5* was also used to improve readability and language of the work. The technology was used with human oversight and control, and all work was reviewed and edited carefully.

## 3 Results

A total of 151 professionals answered the questionnaire between May 2024 and February 2025. Tables 1, 2 present the general characteristics of the respondents and their characteristics in the three professional groups, respectively. There is an apparent imbalance: many advanced medical students responded to the call (hence the high percentage of undergraduate respondents with non-ED medical background), while few students with technical IT background completed the questionnaire.

**Table 1:** General characteristics of the respondents.

*N* = 151 respondents in total	Counts (% of total)
Gender	
male	73 (48.3%)
female	75 (49.7%)
I prefer not to tell	3 (2%)
Professional background	
Medical other than emergency medicine	58 (38.4%)
Emergency medicine	24 (15.9%)
Informatics	19 (12.2%)
Mathematics	‒
Computer engineering	17 (11.3%)
Software engineering	23 (15.2%)
Other not in the above	10 (6.6%)
Level of education & profession	
Ongoing undergraduate studies (1)	61 (40.4%)
Graduate or junior professional (2)	44 (29.1%)
Senior professional (3)	46 (30.5%)

**Table 2:** Characteristics of respondents in the three professional groups.

*N* = 151 respondents in total	*N*	Level of education & profession		
		1—undergraduate	2—junior graduate	3—senior
		Counts (% of line total)	Counts (% of line total)	Counts (% of line total)
Gender				
male	73	16 (21.9%)	28 (38.4%)	29 (39.7%)
female	75	45 (60%)	15 (20%)	15 (20%)
I prefer not to tell	3	0	1 (33.3%)	2 (66.7%)
Medical background	*N* = 82 (54.3% from total)			
med ED	24	3 (12.5%)	11 (45.8%)	10 (41.7%)
med non-ED	58	47 (81%)	6 (10.3%)	5 (8.6%)
Technical background	*N* = 69 (45.7% from total)			
tech IT	59	7 (11.9%)	24 (40.7%)	28 (47.5%)
tech non-IT	9	3 (33.3%)	3 (33.3%)	3 (33.3%)

ED, emergency department; IT, information technology related background.

### 3.1 Clustering based on AI-attitude

Table 3 shows low values for both Cronbach's alpha and the ICC of the GAAIS-negative factor, indicating contradictory or inconsistent opinions regarding this construct. Due to the specifics of this project which targeted two distinct classes of professionals, as opposed to a general audience as the scale's developers had done, we decided to continue with the three scales for AI-attitude: GAAIS-positive, GAAIS-negative, and AIAS-4.

**Table 3:** Reliability and consistency of the scale measurements (*N* = 151 respondents in total).

Scale	No of items	Cronbach's alpha	ICC (95% CI)
GAAIS complete	20	0.564	0.061 (0.040–0.088)
GAAIS positive	12	0.763	0.212 (0.165–0.269)
GAAIS negative	8	0.540	0.128 (0.083–0.184)
AIAS-4	4	0.922	0.747 (0.69–0.798)

AIAS-4, 4-item AI attitude scale; CI, confidence interval; GAAIS, general attitudes towards artificial intelligence scale; ICC, intra-class correlation.

*KMeans* clustering was applied to the standardized feature space. The final model used k = 3 clusters, and two PCA factors (which would account for 84.64% of variance). The mean *silhouette* scores with Euclidean distance as a dissimilarity measure were: 0.496, 0.457, and 0.344 for Clusters 0, 1, and 2, respectively. Half of the respondents belong to Cluster 2, and about a quarter to each of the Clusters 0 and 1.

Each cluster contains a mix of medical and technical professionals from all three levels of career and experience. Table 4 synthesizes these characteristics in numbers, and a qualitative summary based on the attitude scale scores (i.e., AIAS-4, GAAIS-positive, GAAIS-negative).

**Table 4:** Characteristics of the three AI-attitude clusters.

*N* = 151 respondents in total	AI-attitude cluster		
	0—cautious	1—enthusiastic	2—balanced
	*N* = 39	*N* = 35	*N* = 77
Attitude scores (qualitative)			
AIAS-4	low	high	moderate-high
GAAIS-positive	low	moderate-high	moderate-low
GAAIS-negative	moderate-high	moderate-low	moderate
Normalized attitude scores	Mean ± SD	Mean ± SD	Mean ± SD
AIAS-4	0.38 ± 0.15	0.91 ± 0.1	0.73 ± 0.11
GAAIS-positive	0.22 ± 0.01	0.7 ± 0.13	0.29 ± 0.13
GAAIS-negative	0.67 ± 0.15	0.48 ± 0.22	0.6 ± 0.16
Background	Counts (% of cluster total)	Counts (% of cluster total)	Counts (% of cluster total)
medical	20 (51.3%)	15 (42.9%)	47 (61%)
non-medical	19 (48.7%)	20 (57.1%)	30 (39%)
Level of career and experience	Counts (% of cluster total)	Counts (% of cluster total)	Counts (% of cluster total)
1 (ongoing undergraduate studies)	13 (33.3%)	14 (40%)	34 (44.2%)
2 (early career)	12 (30.8%)	7 (20%)	25 (32.5%)
3 (senior professional)	14 (35.9%)	14 (40%)	18 (23.4%)
Insight summary			
Dominant background	Balanced	Technical	Medical
Experience skew	Med students Tech seniors	Junior & senior	Mixed
General AI view	Critical, cautious	Positive, confident	Moderate, thoughtful

AIAS-4, 4-item AI attitude scale; GAAIS, general attitudes towards artificial intelligence scale; SD, standard deviation.

**Figure 2:**
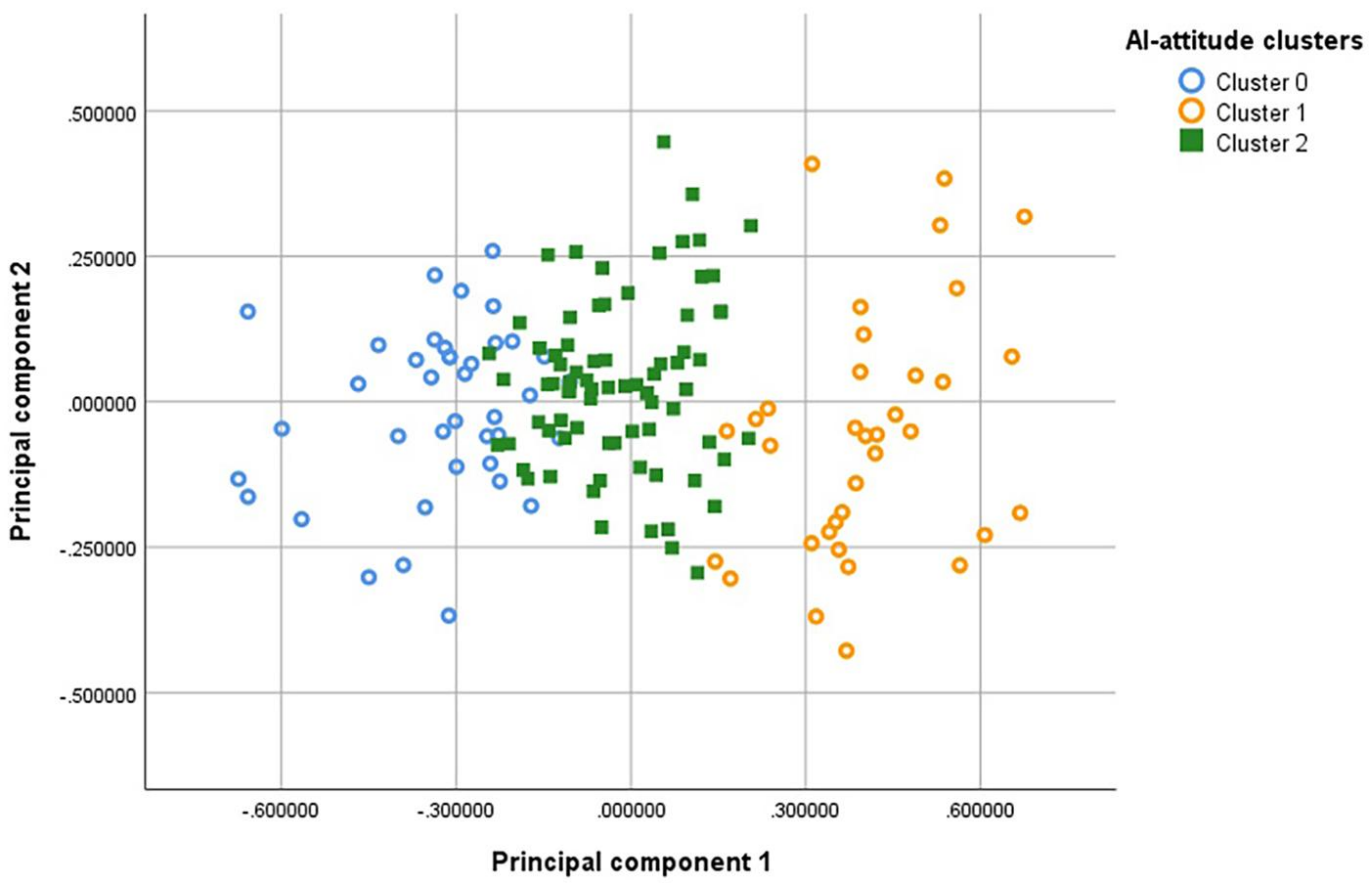
The three AI-attitude clusters of respondents, with the two principal components on the axes.

Figure 2 shows the three clusters with the PCA factors on the axes: Cluster 0 (blue), cautious/ critical attitudes; Cluster 1 (orange), optimistic/ enthusiastic/ supportive attitudes; Cluster 2 (green), balanced/ pragmatic attitudes.

### 3.2 Tasks AI could address in ED triage. AI methods and tools for implementation

**Figure 3:**
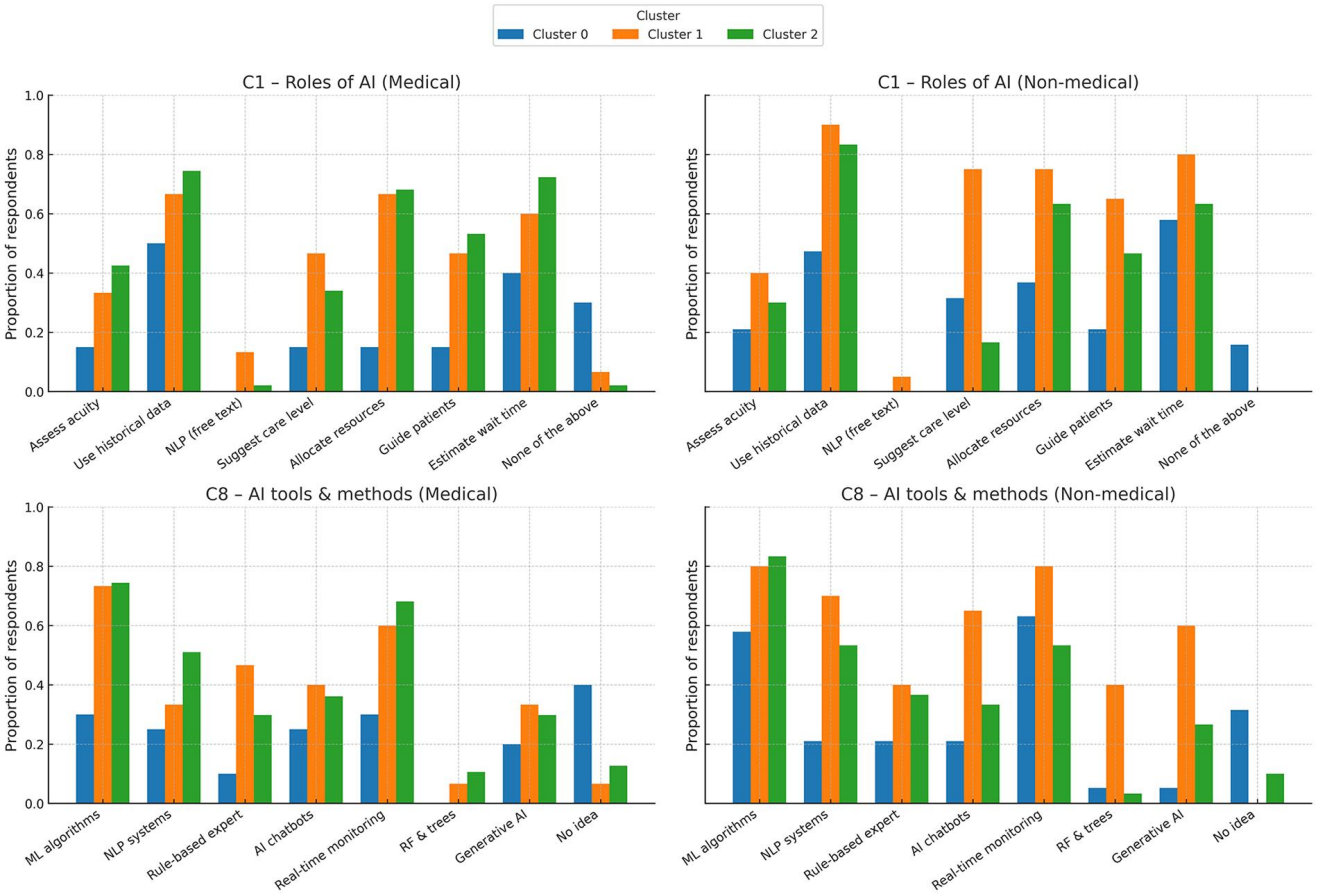
Types of tasks that AI could address in the ED, along with appropriate AI tools or methods to achieve them. AI chatbots, generative AI (such as, ChatGPT, Mistral, Gemini, etc.); ML, machine learning; NLP, natural language processing; RF & Trees, random forest and decision trees.

Question C1 offered a list with potential tasks AI could address (i.e., roles AI applications could play) in optimizing ED triage and patient care; respondents could check as many as they considered feasible, realistic or appropriate. Similarly, question C8 requested opinions on the appropriate AI tools to implement such applications. Figure 3 synthesizes the responses, taking into account medical vs. non-medical background. Machine learning algorithms and real-time monitoring systems are the most widely supported tools across all clusters. Non-medical respondents in Cluster 1 (AI enthusiasts) endorsed almost all tools, including natural language processing systems, and generative AI. Each of them checked at least one task/ role and at least one AI method/ tool. Cluster 0 (AI skeptics)—especially medical professionals—showed limited support and the highest rates of “no ED task AI can address” and “I have NO idea of AI tools and methods”, indicating uncertainty or lack of familiarity.

**Figure 4:**
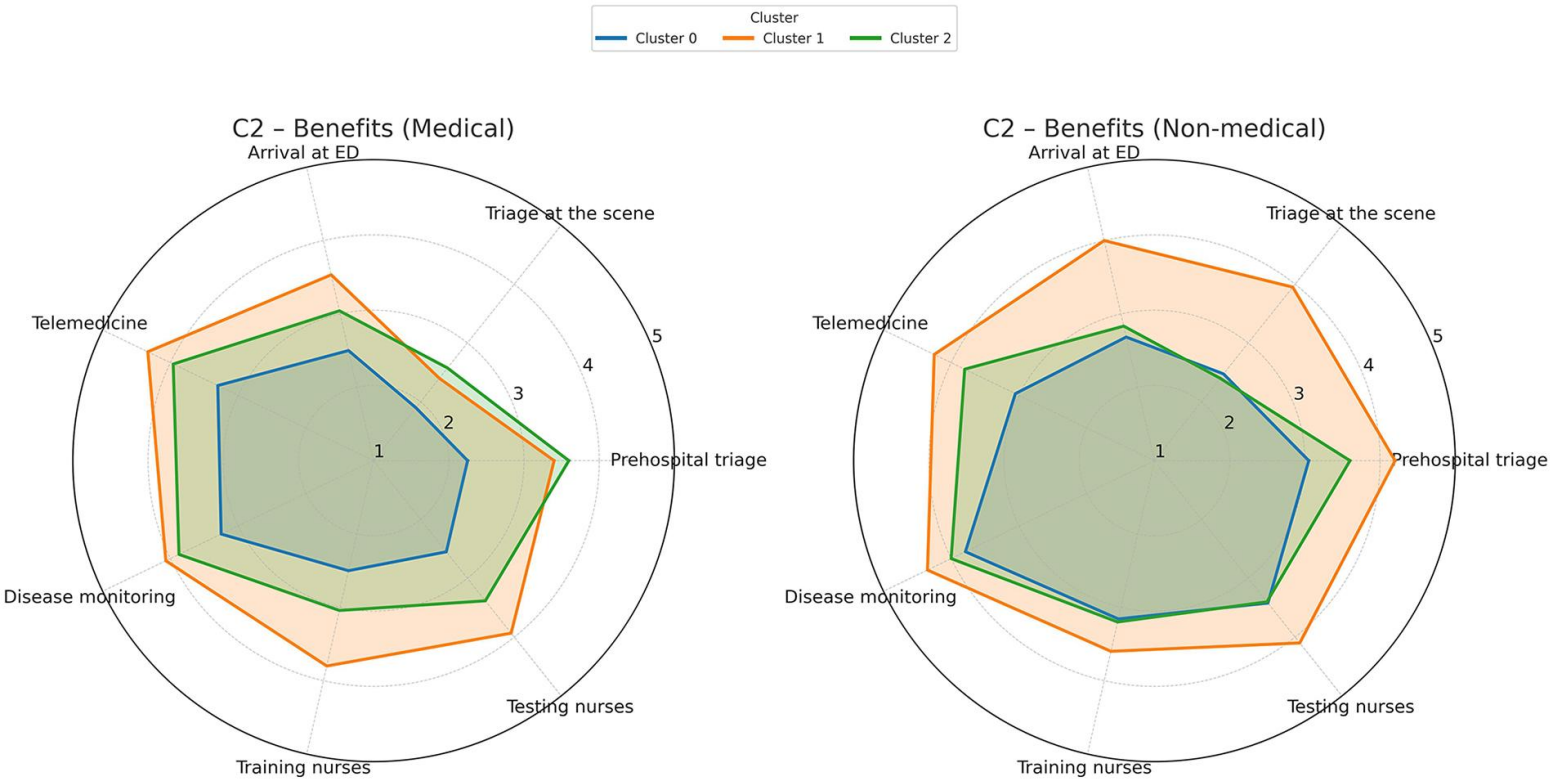
Opinions on the benefits AI could provide in areas such as prehospital triage, on-scene triage, ED arrival triage, telemedicine, disease monitoring, and training/testing of triage nurses. A 5-point Likert scale was used to score the answers.

Figure 4 synthesizes the opinions on levels of benefits AI can provide for various potential employments, such as prehospital triage, triage at the scene of the event, triage upon ED arrival, telemedicine applications, disease monitoring, training and testing the triage nurses. In Cluster 1 (enthusiasts), the contrast between medical and non-medical professionals regarding the triage itself (namely, prehospital triage, triage at the scene of the event, and ED arrival triage) is particularly notable, even enthusiastic medical practitioners being substantially more reserved compared to the technicians.

### 3.3 Constraints and risks

**Figure 5:**
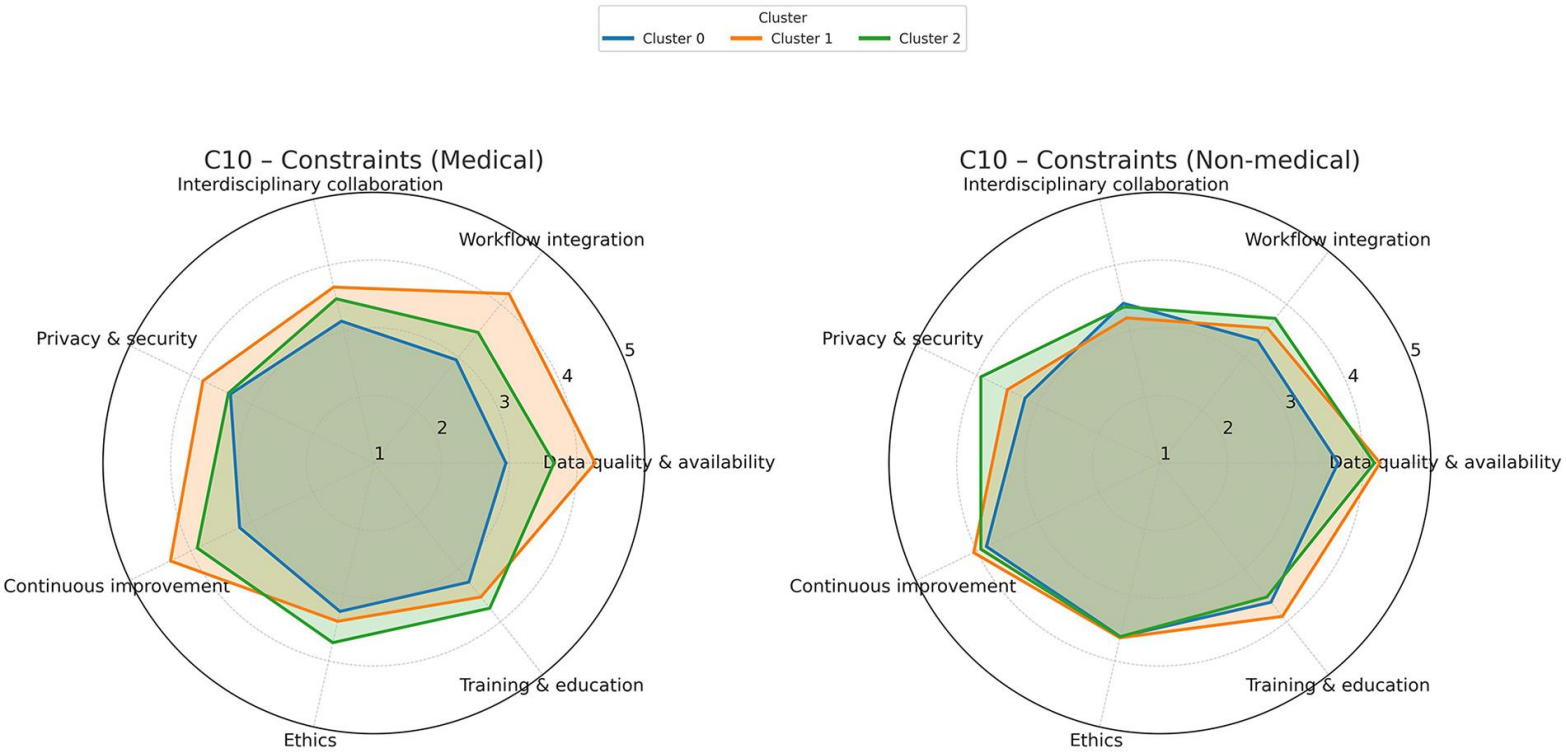
Opinions on constraints needed for effective AI use in triage. All technical professionals recognized their importance. A 5-point Likert scale was used to score the answers.

Figure 5 summarizes opinions on the categories of constraints needed to ensure the effective use of AI in the triage process. All respondents in Cluster 1, both with a medical and a technical background, recognized the importance of imposing such constraints. In contrast, only medical professionals in Cluster 1 (the enthusiasts) emphasized constraints such as data quality, workflow integration, and continuous evaluation and improvement.

**Figure 6:**
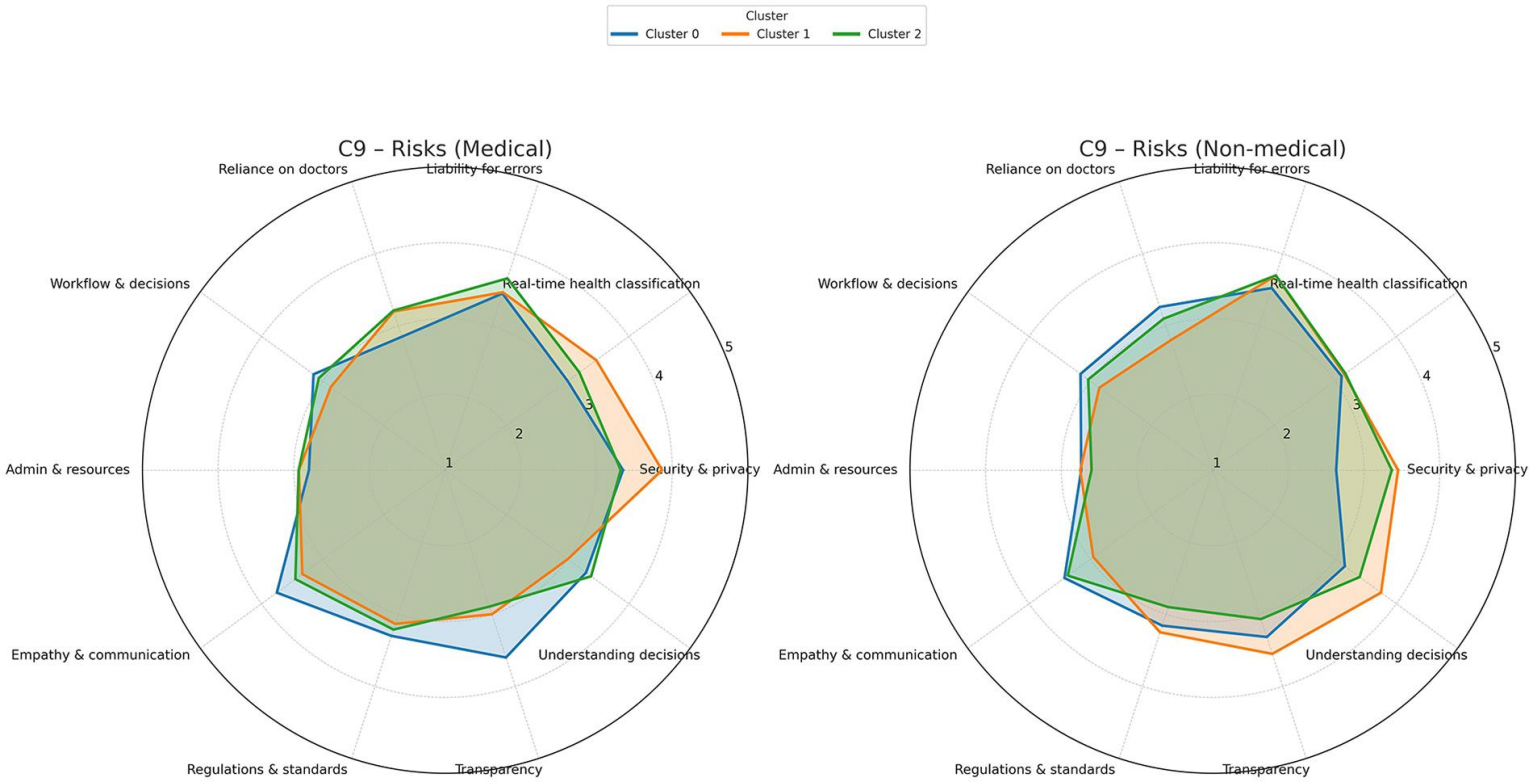
Concerns and risks of AI in emergency medicine. A 5-point Likert scale was used to score the answers.

Figure 6 presents opinions on potential sources of concern and risk associated with AI use in emergency medicine. The misalignments between the clusters, and between professional groups are substantial, although all groups acknowledged some AI-related risks. Medical professionals in Cluster 1 (enthusiasts) expressed substantial concern about data security and privacy, and less concern about AI explainability (namely, the transparency in decision-making and its comprehensibility). Interestingly, medical practitioners in Cluster 2 (the pragmatics) reported few concerns apart from liability and accountability for error. In contrast to all medical professionals, the enthusiastic technicians in Cluster 1 were more concerned about AI explainability, particularly in regard to understanding the decision making.

### 3.4 Metrics of effectiveness and performance

**Figure 7:**
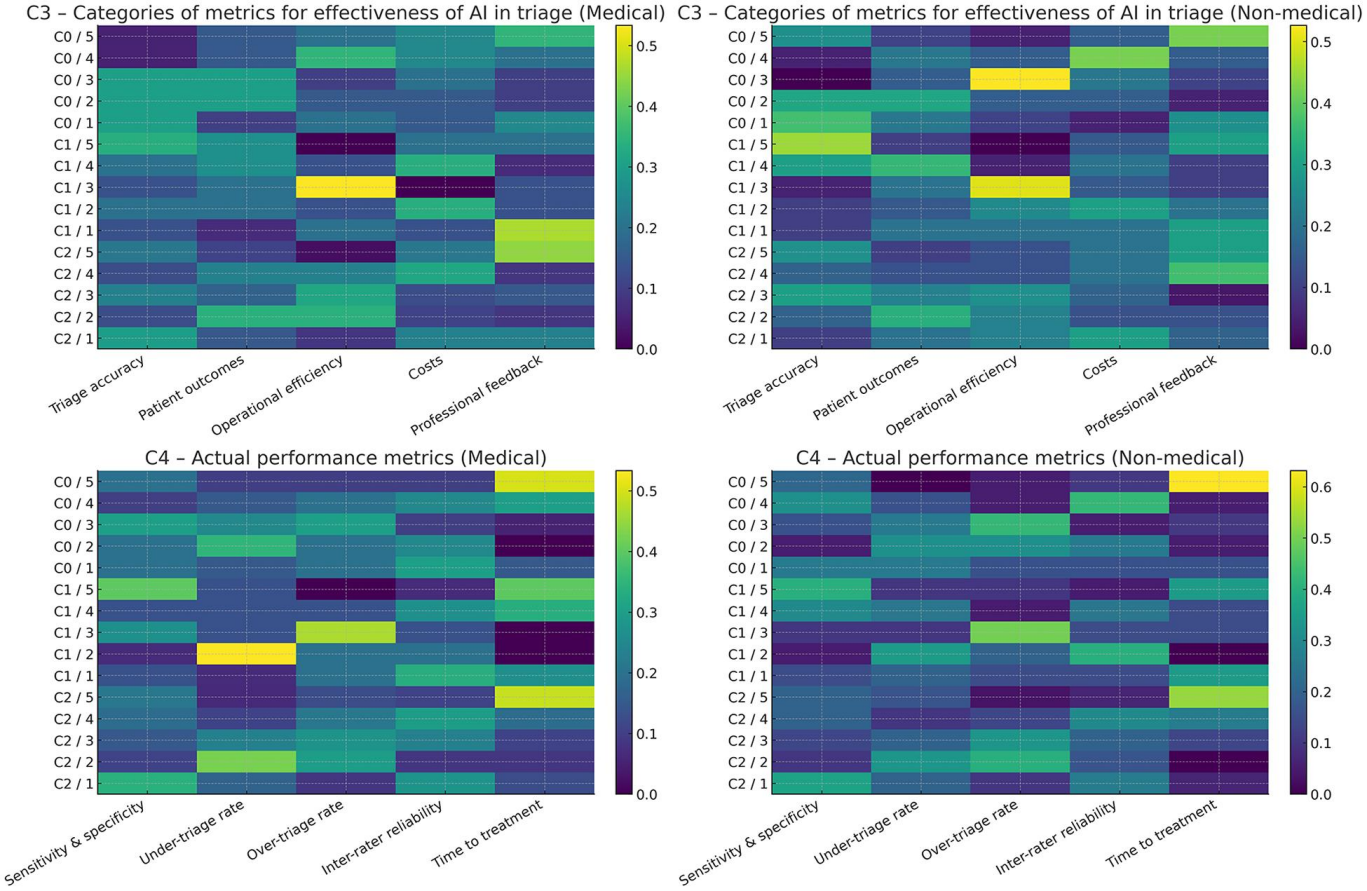
Prioritization of categories and metrics relevant to AI-driven triage in emergency departments. Ranking was enforced in the questionnaire (rank 5 = most important). The clusters (from 0 to 2) and corresponding ranks (from 5 to 1) are on the vertical axes.

Questions C3 and C4 asked the respondents to prioritize the categories of metrics for AI-solutions' effectiveness, and performance (i.e., accuracy) relevant to AI-driven triage in emergency departments, respectively. Figure 7 summarizes their opinions in the form of heatmaps, with rank 5 being the most important. All professionals in Cluster 0 and Cluster 1 emphasized the average importance of operational metrics, while opinions on the other categories varied considerably.

Opinions of the importance of accuracy indicators to assess the success of an AI-driven triage is notably dependent on the respondents' background: a higher proportion of technicians considered it as the key indicator of effectiveness, while most healthcare professionals considered it in balance with the professional feedback.

Based on the prior assumption that triage accuracy is an important category of effectiveness metrics, question C4 aimed at identifying the highest priority metric(s) of accuracy. Background-related variability was less regarding actual triage accuracy, compared to effectiveness in general: “time to treatment” and “sensitivity/specificity” were the most frequently ranked highest criteria, despite still notable variability across clusters (Figure 7).

### 3.5 Social and human factors

**Figure 8:**
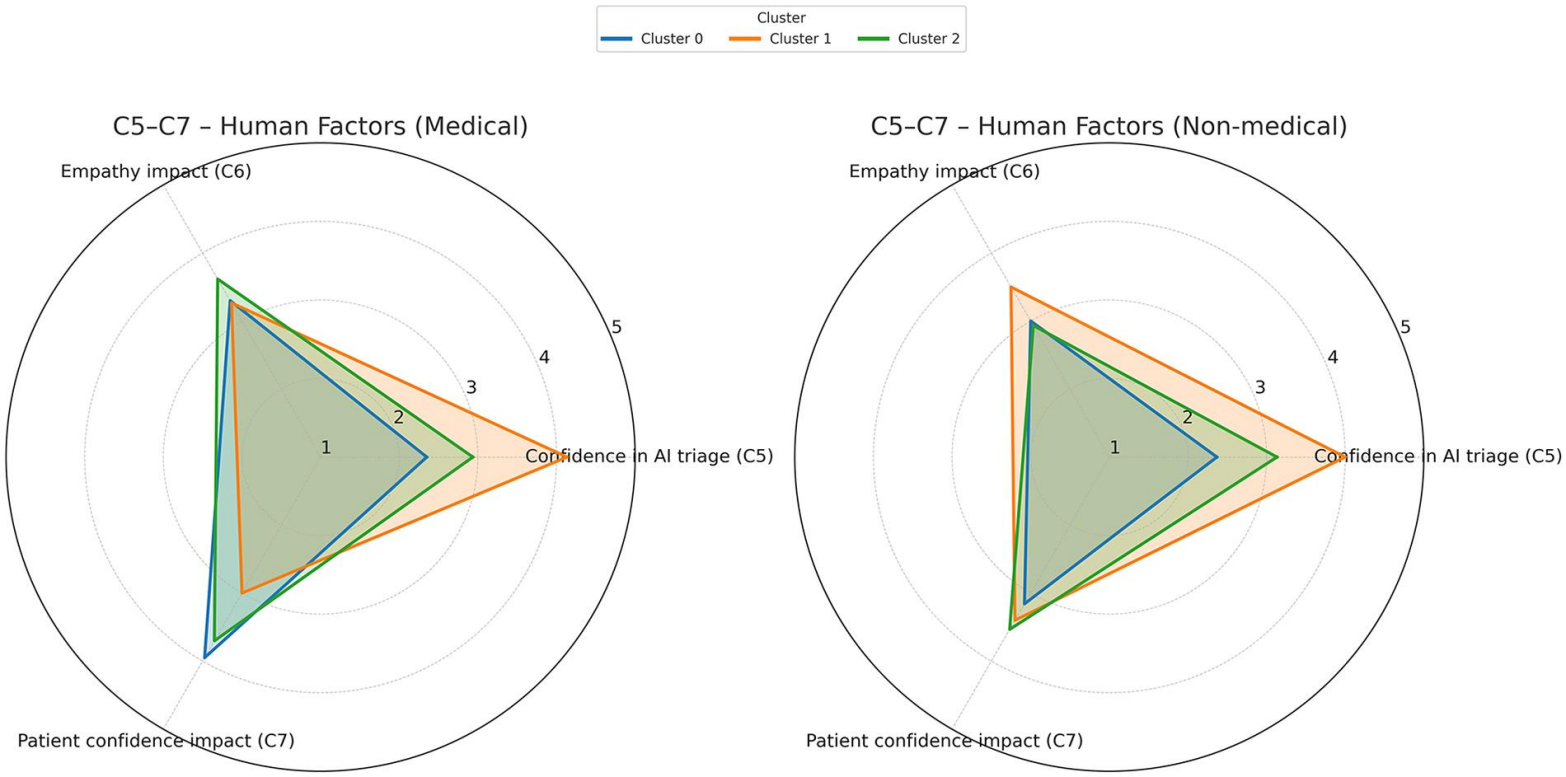
Opinions on confidence in AI systems for ED triage, AI's impact on doctors’ empathy, and patients’ confidence during triage. A 5-point Likert scale was used to score the answers.

Questions C5, C6, and C7 explored respondents' confidence in AI systems for ED triage, perceptions of AI's impact on doctors' empathy towards patients, and patients' confidence in the care received during triage. Figure 8 summarizes these “confidence triangles” for medical and non-medical professionals. Among medical professionals, Cluster 1 (the enthusiasts) showed high trust in AI's triage ability and less concern about empathy impact or patients' confidence, while Cluster 2 (the pragmatics) and Cluster 0 (the cautious) showed higher concern about the impact on the patients’ confidence in the care they receive. All medical professionals expressed concern about the impact on the empathy in doctors' interaction with patients during the triage process. Non-medical professionals followed similar patterns but were generally less worried about patients' confidence than their medical counterparts.

### 3.6 Integration with UTAUT2

Data collected were mapped on the UTAUT2 constructs. Table 5 shows the mapping: GAAIS and AIAS-4 items were searched first. Not all UTAUT2 constructs were retrievable from the data: for example, information regarding the effort expectancy and habit was not collected (mainly because AI is too young a field for a habit to be developed and acknowledged).

**Table 5:** Mapping to UTAUT2 constructs.

UTAUT2 constructs (38, 39)	Definition	Mapped items
Performance expectancy	Belief that using AI will provide benefits (e.g., job performance, outcomes)	GAAIS2, GAAIS5, GAAIS11, GAAIS14, GAAIS17, GAAIS18, AIAS1, AIAS2, AIAS3
Effort expectancy	Ease of use, how effortless using AI feels	Could be implied by AIAS3—not directly measured; was not included in further analysis
Social influence	Perceived social pressure to use or trust AI	GAAIS3, GAAIS6, GAAIS10, GAAIS19, C6, C7
Facilitating conditions	Availability of support, resources, and infrastructure	Items in C10 group (constraints required to ensure effective use of AI in the triage process)
Hedonic motivation	Fun/enjoyment associated with using the technology	GAAIS12, AIAS4
Price value	Cost-benefit perception	C3m4aiCosts, GAAIS2, GAAIS11
Habit	Behavioral tendency from past use	Could be AIAS3 (intention to use AI in the future) but no past use was captured in this study; was not included in further analysis

AIAS-4, 4-item AI attitude scale; C3m4aiCosts, rank of cost analysis as a metric of effectiveness; GAAIS, general attitudes towards artificial intelligence scale; UTAUT2, extended unified theory of acceptance and use of technology.

Table 6 synthesizes the descriptive statistics for these constructs within each AI-attitude cluster. They are meaningful within each construct, but not across them (i.e., they were not normalized). The hedonic motivation appears to make a substantial difference between the clusters; it might also be associated to the cost-benefit perception, which similarly has the highest values in Cluster 0.

**Table 6:** AI-attitude clusters’ insights and their UTAUT2 mapping. The clusters’ colors were used in graphical representations (such as Figures 2–6). Different ranges of values apply for each UTAUT2 construct.

*N* = 151 respondents	Cluster 0 (blue) Cautious	Cluster 1 (orange) Optimistic/enthusiastic	Cluster 3 (green) Pragmatic/balanced
	*N* = 39	*N* = 35	*N* = 77
UTAUT2 constructs (38, 39)	Mean ± SD	Mean ± SD	Mean ± SD
Performance expectancy	1.71 ± 0.19	2.02 ± 0.18	1.86 ± 0.18
Social influence	3.36 ± 1.09	3.31 ± 0.95	3.49 ± 0.85
Facilitating conditions	3.34 ± 0.8	3.8 ± 0.95	3.68 ± 0.8
Hedonic motivation	3.41 ± 1	6.86 ± 0.97	5.07 ± 1.08
Price value	3.38 ± 1.27	2.94 ± 1.41	3.05 ± 1.51

SD, standard deviation; UTAUT2, extended unified theory of acceptance and use of technology.

## 4 Discussions

Our cross-sectional survey collected opinions and attitudes about AI in general, and AI-supported ED triage in particular. One hundred and fifty-one professionals answered the questionnaire: healthcare providers on the one hand, and technicians, mainly IT-related, on the other hand. Irrespective of their background and level of career, the respondents were grouped into three clusters, based on their general attitude towards AI: Cluster 0 (cautious or skeptical); Cluster 1 (generally enthusiastic and supportive); Cluster 2 (pragmatic and implementation-focused). Approximately half of the respondents belong to Cluster 2, of which more than 60% had medical training; more than half of this pragmatic respondents (43/77, 55.9%) were professional graduates, namely early career professionals and senior professionals from both backgrounds.

On many ED triage-related topics, the opinions appeared to be primarily related to the general AI-attitude. Such attitude-based opinions comprised: AI-based solutions would be useful in ED triage to solve problems like assessing the level of acuity and urgency of patients' condition and direct them to appropriate care level, processing historical data of patients, optimizing resource allocation, or estimating the waiting time; anticipated benefits comprise disease monitoring, telemedicine, and testing in education; time to treatment is an important metric to assess the accuracy of ED triage; most AI tools or methods could be employed in AI-driven triage. There were also topics on which opinions were rather determined by the respondents' backgrounds (i.e., medical vs. non-medical): healthcare professionals had notably higher rates of negative opinions of prospective AI-based ED triage design, implementation, and employable methods, i.e., “no ED task AI can address” and “I have no idea of AI tools and methods”, respectively; healthcare providers were more reserved regarding the AI-driven solutions for triage at the scene of an event; medical vs. non-medical respondents showed rather divergent opinions about the constraints and risks associated with AI-supported solutions, and regarding most human factors related to empathy or trust.

Our data showed substantial mis-alignment between medical and non-medical professionals regarding the classes of constraints required to ensure effective use of AI in the triage process and the potential risks associated with such AI-driven systems. Quality and availability of data in healthcare systems is acknowledged only by the enthusiastic (i.e., a quarter) healthcare professionals; this topic has already been raised as a potential issue at an organizational level (5, 15, 17, 32). Moreover, the importance of integration of such a system with existing workflow is only acknowledged by the same enthusiastic medical practitioners who answered our survey.

There was high variability in perspective of the potential risks associated with AI-driven ED triage. For example, surprisingly, the potential lack of explainability in the AI-driven triage was not seen as an issue by the medical professionals in Clusters 1 and 2 (i.e., the enthusiasts and the pragmatics), in contrast to all the technicians. This heterogeneity in perspective may be due to the emergent character of AI-supported systems in everyday clinical practice on the one hand, and to the insufficient educational support in this field on the other hand. Moreover, having AI-confident healthcare professionals is related to building AI-competent healthcare workforce able to participate in internal and external collaboration, therefore the medical education should include the objective of developing AI literacy in addition to data science literacy (3, 4, 26, 30, 44–48). Such AI-relevant learning in medical education should help healthcare professionals understand both the challenges and advantages of AI-driven solutions, understand what to expect from such systems, and bridge the “AI chasm” (2, 6), so fewer would answer “no idea” to questions regarding the AI tools or tasks in ED triage. For example, AI is paramount in natural language and free text processing, and in other related applications such as simulation-based training and testing, and metaverse in ED (1, 12, 15, 49–51), while very few respondents identified this potential in our survey.

The International Medical Informatics Association (IMIA) has updated its recommendations on education in biomedical and health informatics to include AI as a required competence (52). The European Society for Emergency Medicine (EUSEM) has a working group for digital emergency medicine (53), and an agreement with the European Federation for Medical Informatics (EFMI) to collaborate on both research and education (54). Disseminating success stories of significant improvements in triage equity through the effective implementation of AI-supported ED triage (17–20) and specialized training for AI-translators (namely, enthusiastic medical professionals from Cluster 1) would help to establish realistic expectations for interdisciplinary projects involving professionals from various backgrounds, i.e., both developers and consumers of AI-driven solutions in ED triage. Notably, interdisciplinary educational programmes on AI in healthcare already exist and aim to attract students from diverse backgrounds (55, 56). Integrating AI courses into general medical education would help develop AI-based critical thinking skills; establishing dedicated ethical frameworks and application guidelines is recognised as a crucial first step in advancing interdisciplinary approaches to deploying AI-assisted solutions in healthcare more broadly, and in ED triage in particular (26, 47, 48).

There was also heterogeneity and misalignments in the metrics for assessing effectiveness of an AI application in ED triage. As evidence-based medicine is now common practice, the integration of AI-derived evidence is also expected to intensify. Reporting protocols are already in place: TRIPOD-AI and PROBAST-AI (57).

Hedonic motivation seemed to be the optimism driving force among the enthusiasts in our survey, which confirms the investigations regarding healthcare and other fields, such as education (39, 40). It could be related to respondents' trust and familiarity with AI tools.

In summary, there is an on-going re-negotiation regarding the role AI could play in emergency triage (in relation to the means of implementation, challenges, and constraints) on the one hand, and the role of emergency physicians and their responsibilities on the other hand. While most professionals are rather moderate and balanced, there is great heterogeneity in the vision of developers and consumers, and across the levels of professional experience. AI-driven solutions in healthcare are emerging, subjective opinions are contradictory, and numerous confounding factors constitute additional obstacles to the development of a coherent vision. Multidisciplinary educational programmes on AI in healthcare are needed to develop the knowledge of bridging such existing gaps and mitigating the present contradictions. Trained enthusiasts from both medical and technical backgrounds can play an important role as AI-translators, advancing the field without compromising confidence in medical decision-making.

### 4.1 Limitations

The cross-sectional design and the number of respondents are the main limitations to the survey's validity, but there are underlying reasons for assuming these shortcomings. Firstly, this is a highly dynamic field; only an analysis over a limited period of time could provide a realistic picture of it, with its inherent limitations. Our investigation aims to put forward the subject to the attention of educators, policy makers, and leaders for future investigation. Secondly, the survey's distribution to the targeted English-speaking professionals was intentionally limited so we could ask domain-specific questions and contain the confounding factors.

The reliability and consistency of the GAAIS scale was low to moderate, particularly for factor GAAIS-negative; this would imply rather inconsistent negative perceptions and concerns regarding AI-related risks. They could be due to various factors, such as the heterogeneity in professional background or level of career, and individual psychological traits (which were not separately assessed in our exploratory investigation). This moderate inconsistency affected the clustering quality (i.e., the clusters were not disjoint), but the mean *silhouette* scores remained acceptable.

The mix of medical professionals who answered the questionnaire−ED and non-ED physicians−could also be regarded as a limitation. In addition, it was an anonymous and voluntary questionnaire, which did not allow for verification of factual information (e.g., background and level of career) or assessment of the level of professional knowledge or understanding of AI concepts. However, our investigation aimed to explore subjective opinions and attitudes, rather than synthesizing a professional report. Moreover, very often, medical practitioners need assistance of AI-based tools to work or practice beyond their strict expertise, for example when they face difficulties and need support; to use these AI-driven systems, they should both trust them and have the necessary know-how. Furthermore, the actual clusters did not show distinguishable differences in terms of professional background or career level; even with regard to attitude towards AI, although *silhouette* scores indicated acceptable clustering, the actual clusters were not disjoint, with a notably lower silhouette score for Cluster 2, i.e., the thoughtful, moderate half of respondents. On the one hand, the anonymous character of the questionnaire constitutes a limitation or shortcoming; on the other hand, it offers the advantage of avoiding the drawbacks associated with interviews and identifiable responses, such as selection bias, social desirability bias, and acquiescence bias.

This was an exploratory investigation and no hypotheses were tested. Our analysis provides no definite answers, but they are not actually needed in this highly dynamic developing field. UTAUT2 mapping is also merely exploratory and was not validated; therefore, no normalization was applied to compare the contribution of the UTAUT2 constructs to different opinions regarding ED triage; in addition, some GAAIS items are not related to the UTAUT2 theory, but rather to psychological components of the attitude towards AI.

## 5 Conclusions

AI-based solutions for optimizing ED triage should be analyzed beyond the performance of the algorithms. There is substantial misalignment in perspective of various stakeholders. This depends only to a certain extent on background and potential roles as developers or consumers. Even among professionals with similar background and expertise, hedonic motivation plays an important role in driving the interest in AI applications. Two educational directions should be followed: (a) AI courses and comprehensive programs should target the enthusiastic developers from diverse backgrounds; (b) all healthcare professionals should acquire AI literacy and become competent consumers.

## Data Availability

The datasets presented in this study can be found on figshare: https://doi.org/10.6084/m9.figshare.30847343.

## References

[1] Maslej N Fattorini L Perrault R Gil Y Parli V Kariuki N The AI Index 2025 Annual Report. Stanford, CA: Institute for Human-Centered AI, Stanford University (2025). 10.48550/arXiv.2504.07139

[2] Tikhomirov L Semmler C McCradden M Searston R Ghassemi M Oakden-Rayner L. Medical artificial intelligence for clinicians: the lost cognitive perspective. Lancet Digit Health. (2024) 6:e589–94. 10.1016/S2589-7500(24)00095-5 39059890

[3] Liu F Zhou H Wang K Yu Y Gao Y Sun Z MetaGP: a generative foundation model integrating electronic health records and multimodal imaging for addressing unmet clinical needs. Cell Rep Med. (2025) 6:102056. 10.1016/j.xcrm.2025.102056 40187356 PMC12047458

[4] Patil R Heston TF Bhuse V. Prompt engineering in healthcare. Electronics (Basel). (2024) 13:2961. 10.3390/electronics13152961

[5] Ranjbar A Mork E Ravn J Brøgger H Myrseth P Østrem HP Managing risk and quality of AI in healthcare: are hospitals ready for implementation? Risk Manag Healthc Policy. (2024) 17:877–882. 10.2147/RMHP.S452337 38617593 PMC11016246

[6] Seneviratne MG Shah NH Chu L. Bridging the implementation gap of machine learning in healthcare. BMJ Innov. (2020) 6:45–47. 10.1136/bmjinnov-2019-000359

[7] Zachariasse JM van der Hagen V Seiger N Mackway-Jones K van Veen M Moll HA. Performance of triage systems in emergency care: a systematic review and meta-analysis. BMJ Open. (2019) 9:e026471. 10.1136/bmjopen-2018-026471 31142524 PMC6549628

[8] Tam HL Chung SF Lou CK. A review of triage accuracy and future direction. BMC Emerg Med. (2018) 18:58. 10.1186/s12873-018-0215-0 30572841 PMC6302512

[9] Hinson JS Martinez DA Cabral S George K Whalen M Hansoti B Triage performance in emergency medicine: a systematic review. Ann Emerg Med. (2019) 74:140–152. 10.1016/j.annemergmed.2018.09.022 30470513

[10] Lansiaux E Ibrahim E Minaz A Itani R Kukkadi R Obinna O Medical triage: from battlegrounds darkness to the machine light. Soc Sci Res Netw (SSRN). (2024). 10.2139/ssrn.4713040

[11] Levine DM Tuwani R Kompa B Varma A Finlayson SG Mehrotra A The diagnostic and triage accuracy of the GPT-3 artificial intelligence model: an observational study. Lancet Digit Health. (2024) 6:e555–61. 10.1016/S2589-7500(24)00097-9 39059888

[12] Salman OH Taha Z Alsabah MQ Hussein YS Mohammed AS Aal-Nouman M. A review on utilizing machine learning technology in the fields of electronic emergency triage and patient priority systems in telemedicine: coherent taxonomy, motivations, open research challenges and recommendations for intelligent future work. Comput Methods Programs Biomed. (2021) 209:106357. 10.1016/j.cmpb.2021.106357 34438223

[13] Le K Chen J Mai D Le KDR. An evaluation on the potential of large language models for use in trauma triage. Emerg Care Med. (2024) 1:350–67. 10.3390/ecm1040035

[14] Fernandes M Vieira SM Leite F Palos C Finkelstein S Sousa JMC. Clinical decision support systems for triage in the emergency department using intelligent systems: a review. Artif Intell Med. (2020) 102:101762. 10.1016/j.artmed.2019.101762 31980099

[15] Chenais G Lagarde E Gil-Jardiné C. Artificial intelligence in emergency medicine: viewpoint of current applications and foreseeable opportunities and challenges. J Med Internet Res. (2023) 25:e40031. 10.2196/40031 36972306 PMC10245226

[16] Porto BM. Improving triage performance in emergency departments using machine learning and natural language processing: a systematic review. BMC Emerg Med. (2024) 24:219. 10.1186/s12873-024-01135-2 39558255 PMC11575054

[17] Da’Costa A Teke J Origbo JE Osonuga A Egbon E Olawade DB. AI-driven triage in emergency departments: a review of benefits, challenges, and future directions. Int J Med Inform. (2025) 197:105838. 10.1016/j.ijmedinf.2025.105838 39965433

[18] Taylor RA Chmura C Hinson J Steinhart B Sangal R Venkatesh AK Impact of artificial intelligence–based triage decision support on emergency department care. NEJM AI. (2025) 2(3):AIoa2400296. 10.1056/AIoa2400296

[19] Levin S Toerper M Hinson J Gardner H Henry S McKenzie C 294 machine-learning-based electronic triage: a prospective evaluation. Ann Emerg Med. (2018) 72:S116. 10.1016/j.annemergmed.2018.08.299 28888332

[20] Hinson JS Levin SR Steinhart BD Chmura C Sangal RB Venkatesh AK Enhancing emergency department triage equity with artificial intelligence: outcomes from a multisite implementation. Ann Emerg Med. (2025) 85:288–290. 10.1016/j.annemergmed.2024.10.014 39570253

[21] Batko K Ślęzak A. The use of big data analytics in healthcare. J Big Data. (2022) 9:3. 10.1186/s40537-021-00553-4 35013701 PMC8733917

[22] Colakca C Ergın M Ozensoy HS Sener A Guru S Ozhasenekler A. Emergency department triaging using ChatGPT based on emergency severity index principles: a cross-sectional study. Sci Rep. (2024) 14:22106. 10.1038/s41598-024-73229-7 39333599 PMC11436771

[23] Masanneck L Schmidt L Seifert A Kölsche T Huntemann N Jansen R Triage performance across large language models, ChatGPT, and untrained doctors in emergency medicine: comparative study. J Med Internet Res. (2024) 26:e53297. 10.2196/53297 38875696 PMC11214027

[24] Riboli-Sasco E El-Osta A Alaa A Webber I Karki M El Asmar ML Triage and diagnostic accuracy of online symptom checkers: systematic review. J Med Internet Res. (2023) 25:e43803. 10.2196/43803 37266983 PMC10276326

[25] Hoppe JM Auer MK Strüven A Massberg S Stremmel C. ChatGPT with GPT-4 outperforms emergency department physicians in diagnostic accuracy: retrospective analysis. J Med Internet Res. (2024) 26:e56110. 10.2196/56110 38976865 PMC11263899

[26] Ng FYC Thirunavukarasu AJ Cheng H Tan TF Gutierrez L Lan Y Artificial intelligence education: an evidence-based medicine approach for consumers, translators, and developers. Cell Rep Med. (2023) 4:101230. 10.1016/j.xcrm.2023.101230 37852174 PMC10591047

[27] Ahun E Demir A Yiğit Y Tulgar YK Doğan M Thomas DT Perceptions and concerns of emergency medicine practitioners about artificial intelligence in emergency triage management during the pandemic: a national survey-based study. Front Public Health. (2023) 11:1285390. 10.3389/fpubh.2023.1285390 37965502 PMC10640989

[28] Scott IA Carter SM Coiera E. Exploring stakeholder attitudes towards AI in clinical practice. BMJ Health Care Inform. (2021) 28:e100450. 10.1136/bmjhci-2021-100450 34887331 PMC8663096

[29] Čartolovni A Malešević A Poslon L. Critical analysis of the AI impact on the patient–physician relationship: a multi-stakeholder qualitative study. Digit Health. (2023) 9:1–14. 10.1177/20552076231220833 PMC10734361 38130798

[30] Petersson L Larsson I Nygren JM Nilsen P Neher M Reed JE Challenges to implementing artificial intelligence in healthcare: a qualitative interview study with healthcare leaders in Sweden. BMC Health Serv Res. (2022) 22:850. 10.1186/s12913-022-08215-8 35778736 PMC9250210

[31] Stewart J Freeman S Eroglu E Dumitrascu N Lu J Goudie A Attitudes towards artificial intelligence in emergency medicine. Emerg Med Australas. (2024) 36:252–65. 10.1111/1742-6723.14345 38044755

[32] Townsend BA Plant KL Hodge VJ Ashaolu O Calinescu R. Medical practitioner perspectives on AI in emergency triage. Front Digit Health. (2023) 5:1297073. 10.3389/fdgth.2023.1297073 38125759 PMC10731272

[33] Schepman A Rodway P. Initial validation of the general attitudes towards artificial intelligence scale. Comput Human Behav Rep. (2020) 1:100014. 10.1016/j.chbr.2020.100014 PMC7231759 34235291

[34] Schepman A Rodway P. The general attitudes towards artificial intelligence scale (GAAIS): confirmatory validation and associations with personality, corporate distrust, and general trust. Int J Hum Comput Interact. (2023) 39:2724–41. 10.1080/10447318.2022.2085400

[35] Grassini S. Development and validation of the AI attitude scale (AIAS-4): a brief measure of general attitude toward artificial intelligence. Front Psychol. (2023) 14:1191628. 10.3389/fpsyg.2023.1191628 37554139 PMC10406504

[36] Talik W Talik EB Grassini S. Measurement invariance of the artificial intelligence attitude scale (AIAS-4): cross-cultural studies in Poland, the USA, and the UK. Curr Psychol. (2025) 44:15758–66. 10.1007/s12144-025-08348-z

[37] Podsakoff PM MacKenzie SB Lee J-Y Podsakoff NP. Common method biases in behavioral research: a critical review of the literature and recommended remedies. J Appl Psychol. (2003) 88:879–903. 10.1037/0021-9010.88.5.879 14516251

[38] Venkatesh TX. Consumer acceptance and use of information technology: extending the unified theory of acceptance and use of technology. MIS Q. (2012) 36:157. 10.2307/41410412

[39] Venkatesh V Thong J Xu X. Unified theory of acceptance and use of technology: a synthesis and the road ahead. J Assoc Inf Syst. (2016) 17:328–376. 10.17705/1jais.00428

[40] Tamilmani K Rana NP Wamba SF Dwivedi R. The extended unified theory of acceptance and use of technology (UTAUT2): a systematic literature review and theory evaluation. Int J Inf Manage. (2021) 57:102269. 10.1016/j.ijinfomgt.2020.102269

[41] Marikyan D Papagiannidis S Stewart G. Technology acceptance research: meta-analysis. J Inf Sci. (2023) 0(0):1–22. 10.1177/01655515231191177

[42] Al Mubarak M Hamdan A. Innovative and intelligent digital technologies; towards an increased efficiency. In: Al Mubarak M Hamdan A, editors. Cham: Springer Nature Switzerland. (2024) ISBN 978-3-031-71649-2 (eBook). 10.1007/978-3-031-71649-2

[43] Rousseeuw PJ. Silhouettes: a graphical aid to the interpretation and validation of cluster analysis. J Comput Appl Math. (1987) 20:53–65. 10.1016/0377-0427(87)90125-7

[44] Lungeanu D Petrica A Lupusoru R Marza AM Mederle OA Timar B. Beyond the digital competencies of medical students: concerns over integrating data science basics into the medical curriculum. Int J Environ Res Public Health. (2022) 19:15958. 10.3390/ijerph192315958 36498065 PMC9739359

[45] Acosta-Enriquez BG Ramos Farroñan EV Villena Zapata LI Mogollon Garcia FS Rabanal-León HC Angaspilco JEM Acceptance of artificial intelligence in university contexts: a conceptual analysis based on UTAUT2 theory. Heliyon. (2024) 10:e38315. 10.1016/j.heliyon.2024.e38315 39430455 PMC11489141

[46] Brommeyer M Whittaker M Mackay M Ng F Liang Z. Building health service management workforce capacity in the era of health informatics and digital health—a scoping review. Int J Med Inform. (2023) 169:104909. 10.1016/j.ijmedinf.2022.104909 36347141

[47] Rodger D Mann SP Earp B Savulescu J Bobier C Blackshaw BP. Generative AI in healthcare education: how AI literacy gaps could compromise learning and patient safety. Nurse Educ Pract. (2025) 87:104461. 10.1016/j.nepr.2025.104461 40633198

[48] Li X Yan X Lai H. The ethical challenges in the integration of artificial intelligence and large language models in medical education: a scoping review. PLoS One. (2025) 20:e0333411. 10.1371/journal.pone.0333411 41124146 PMC12543126

[49] Wu T-C Ho C-TB. A scoping review of metaverse in emergency medicine. Australas Emerg Care. (2023) 26:75–83. 10.1016/j.auec.2022.08.002 35953392

[50] Chang Y-H Lin Y-C Huang F-W Chen D-M Chung Y-T Chen W-K Using machine learning and natural language processing in triage for prediction of clinical disposition in the emergency department. BMC Emerg Med. (2024) 24:237. 10.1186/s12873-024-01152-1 39695961 PMC11657801

[51] Stewart J Lu J Goudie A Arendts G Meka SA Freeman S Applications of natural language processing at emergency department triage: a narrative review. PLoS One. (2023) 18:e0279953. 10.1371/journal.pone.0279953 38096321 PMC10721204

[52] Bichel-Findlay J Koch S Mantas J Abdul SS Al-Shorbaji N Ammenwerth E Recommendations of the international medical informatics association (IMIA) on education in biomedical and health informatics: second revision. Int J Med Inform. (2023) 170:104908. 10.1016/j.ijmedinf.2022.104908 36502741

[53] EUSEM. Working Group Digital Emergency Medicine. Available online at: https://eusem.org/sections-and-committees/working-groups/working-group-digital-emergency-medicine (Accessed November 29, 2025).

[54] EUSEM. EFMI and EUSEM Cooperate to Advance the Digital Transformation of Emergency Departments. Available online at: https://www.eusem.org/about-us/emergency-medicine/what-is-em/290-efmi-and-eusem-cooperate-to-advance-the-digital-transformation-of-emergency-departments (Accessed November 29, 2025).

[55] Imperial College London. AI in Healthcare: Leading Responsible Adoption at Scale. Available online at: https://execed-online.imperial.ac.uk/ai-in-healthcare (Accessed November 30, 2025).

[56] COURSERA, Stanford University. AI in Healthcare Specialization. Available online at: https://www.coursera.org/specializations/ai-healthcare/ (Accessed November 30, 2025).

[57] Collins GS Dhiman P Andaur Navarro CL Ma J Hooft L Reitsma JB Protocol for development of a reporting guideline (TRIPOD-AI) and risk of bias tool (PROBAST-AI) for diagnostic and prognostic prediction model studies based on artificial intelligence. BMJ Open. (2021) 11:e048008. 10.1136/bmjopen-2020-048008 34244270 PMC8273461

